# Male Investment in Nuptial Gifts in *Pisaura mirabilis* (Clerck, 1757) Differs Between Light Conditions

**DOI:** 10.3390/insects16030256

**Published:** 2025-03-02

**Authors:** Pavol Prokop, Zuzana Provazník

**Affiliations:** 1 Department of Environmental Ecology and Landscape Management, Faculty of Natural Sciences, Comenius University, 842 15 Bratislava, Slovakia; zuzana.provaznik@uniba.sk; 2 Institute of Zoology, Slovak Academy of Sciences, 845 06 Bratislava, Slovakia

**Keywords:** *Pisaura mirabilis*, spiders, visual environment, courtship behaviour

## Abstract

Sexual signalling is influenced by environmental conditions, but limited focus has been given to the influence of light conditions on mating strategies in spiders. The males of *Pisaura mirabilis* (Clerck, 1757) produce nuptial gifts by wrapping the dead prey with silk, and female mate choice relies on complex signals involving visual, behavioural, vibratory, and chemical cues. Considering the former, we first examined the natural illumination conditions experienced by *Pisaura mirabilis* in their natural habitat. Specifically, we compared the light levels of spiders situated on leaves to those found within the denser vegetation where they typically reside. In the laboratory, we manipulated the visual conditions during courtship and mating using white (high visibility) and red (low visibility) light, and we blocked male spinnerets with dental silicone to assess the impact of males’ ability to produce silk on mating success. The aim of the study was to examine the effect of visual conditions on male investment in nuptial feeding. The places where spiders spend time outside of vegetation in the wild were 40 times brighter than habitats inside vegetation. The mating success was not significantly dependent on light conditions, but males under red lights invested more effort in the production of nuptial gifts than males under white lights. Males with blocked spinnerets had a lower mating success than silk-intact males. The blocked males under red light, although unable to produce silk, also invested more time in attempts to wrap the gift with silk compared to the silk-intact males or silk-blocked males under white lights. These results suggest that gift production is influenced by an interplay between female behaviour and light conditions, highlighting the significance of taking into account visual conditions in spider mating behaviour research.

## Simple Summary

This study explores how light conditions influence mating strategies in the nursery web spider *Pisaura mirabilis*. Males produce nuptial gifts by wrapping dead prey in silk, and female mate choice relies on various signals. We compared natural light levels in habitats outside and inside dense vegetation, finding that outside areas were 40 times brighter. In the lab, we manipulated light conditions (white and red) during courtship and blocked male spinnerets to assess the impact on mating success. Results showed that while light conditions did not significantly affect mating success, males under red light invested more effort in gift production. Blocked males had lower mating success but still attempted to wrap gifts longer under red light than silk-intact males under white light. These findings highlight the importance of considering visual conditions in spider mating behaviour research.

## 1. Introduction

Sexual selection by mate choice includes a communication component, where the advertising sex is signalling information, and the choosing sex is receiving it [1]. This requires signals that match the sensory systems, and many sexually selected traits, such as colour patterns, are detected and perceived by the visual system [2,3,4]. However, the choosing sex is not merely a passive receiver; they may also emit signals in response to the advertising sex, contributing to the coevolution of signalling traits in both sexes [5]. In complex signals, multiple sensory information may be necessary for signal processing [6].

Visual signals play a crucial role in sexual selection, and the efficacy of these signals is strongly influenced by the visual environment. The transmission properties of the environment can significantly impact the conspicuousness of visual signals and the quality of communication between the signaller and receiver [7,8,9,10]. For example, light intensity, which can vary greatly from 100,000 lux on a sunny day to 0.05 lux on a full moon [11,12], is a key factor regulating the copulatory behaviour of insects [13,14,15] and influences the appearance of animal colour signals [16,17,18].

Variation in light environments within a habitat can cause associated changes in the perceived colour signal parameters, as the spectral composition of the light arriving at the receiver’s eyes will change under different light conditions [9,19,20,21]. This is especially crucial for species in which males frequently encounter diverse environmental conditions [22,23]. In such scenarios, natural selection may favour male displays that effectively convey information across various environments [24]. Mating success relies on the effective communication of information from the signaller to the receiver [25]. Not surprisingly, light conditions shape various aspects of courtship and mating behaviours to optimise individuals’ fitness. For example, Endler [9] hypothesised that females’ ability to select mates during courtship displays is improved at higher light intensities, where males become more conspicuous and their colours more distinguishable. Research has shown that male guppies adjust their display distances based on light intensity [26], with males courting less and using visually conspicuous elements less frequently in the presence of predators or under high light levels [8,9]. Similarly, males of the threadtail damselfly *Protoneura amatoria* employ a hovering mating tactic under low light conditions, where the risk of predation is higher, and a perching mating tactic under high light conditions, when predation risk is lower [27]. When courting females in open areas outside the nest, certain jumping spiders employ visual signals and elevated postures to enhance their display. However, inside the nest, where visibility is low, the males shift to using vibratory displays [28,29]. In a study simulating dark conditions using red light, male wolf spiders with augmented visual cues (enhanced signals that improve their visibility to females) were able to attract females more quickly than in full light, leading to higher mating success rates [30]. In contrast, when the same male cues were presented under white light, which was perceived as normal light conditions by the spiders [31], the time it took males and females to make contact increased, and mating success decreased. Additionally, male wolf spiders (*Rabidosa rabida*) exhibited reduced courtship rates in experimentally simulated darkness compared to those in light conditions. However, when given the opportunity to communicate through seismic signals, their mating rates increased, diminishing the significance of light conditions [23]. This suggests that male courtship is behaviourally plastic, and visual conditions influence male mating success. The interplay between these different sensory channels indicates that communication is multimodal, enhancing mating success—especially in varying environmental conditions where one mode of communication may be compromised.

The courtship behaviour of nursery web spider *Pisaura mirabilis* (Araneae, Pisauridae) males has emerged as a model system for understanding the evolution of nuptial feeding [32,33,34,35]. During courtship, the male constructs a nuptial gift by wrapping dead arthropod prey with silk and offering it to the female [36,37,38,39]. Males rejected by females during courtship frequently add silk to their gift before re-offering, which increases male acceptance [33,40]. Given that gift construction is energy-consuming for males [41], males tend to optimise their investment in silk by carefully allocating their resources to maximise reproductive success while minimising excessive energy expenditure. Despite the recognised importance of nuptial gifts, which are nutritional offerings provided by males to attract females in a mating strategy [42], limited attention has been given to how abiotic factors, particularly light conditions, influence nuptial feeding. Understanding how varying light levels affect male investment in nuptial gifts is important in species where these gifts are integral to visual courtship displays [40,43,44]. Changes in light conditions can influence visibility, thereby affecting female perception and acceptance of the gifts.

One line of evidence suggests that gift acceptance by the female is facilitated by silk-borne chemicals released by males when wrapping the gift in silk. Another line of evidence suggests that gifts painted extra white with watercolour were accepted more quickly, however, than natural gifts or gifts painted with brown [40], indicating that visual conditions play a significant role in gift acceptance. *P. mirabilis* is a diurnal predator that hunts visually [39] and likely has good vision, which is essential for its hunting strategy; its eyes are arranged in two rows, enhancing its ability to perceive the environment and locate prey. Previous research indicates that gift wrapping may play a minor role in the mating success of male *Pisaura mirabilis* under daylight conditions [33,45,46]. This suggests that while wrapped gifts may enhance male attractiveness, the immediate availability of unwrapped prey can still lead to successful mating outcomes. Our approach assumes that silk investment is a plastic trait that varies with male condition; previous research has shown that males in poor condition invest less in silk production than those in good condition [47]. By examining how these various behavioural variables interact with visual conditions and silk investment strategies, we aim to provide a comprehensive understanding of their impact on mating success. In the present study, we noted that males unable to produce silk would offer unwrapped prey.

To test our hypothesis regarding the influence of illuminance on female acceptance of nuptial gifts, we first measured the light levels experienced by *P*. *mirabilis* outside of vegetation during the morning hours and compared them with the light conditions inside vegetation, where these spiders typically reside. Second, we conducted an experiment with a 2 × 2 factorial design, manipulating two factors: visual conditions (good visual conditions with white light and poor visual conditions with red light) and male silk status (silk-blocked males and silk-intact males). Blocking male spinnerets allowed us to control silk production, which is an important part of the courtship behaviour of *P. mirabilis*. In our experiment, we prevented silk production to investigate how male *Pisaura mirabilis* adjust their investment in silk under different visual conditions. We hypothesised that males capable of producing silk would reduce their investment under good visual conditions (white light) because the visibility of their gifts is sufficient for attracting females. Conversely, under poor visual conditions (red light), males would be expected to increase their silk investment to enhance gift visibility, compensating for the reduced effectiveness of their visual signals. Silk-blocked males, unable to produce silk, were anticipated to have mating success similar to that of intact males under white light, as the visual clarity negates the need for additional silk. However, when silk production was prevented in poor visual conditions, these males were expected to exhibit the lowest mating success due to the diminished visibility of their gifts.

## 2. Materials and Methods

### 2.1. Study Organism

The nursery web spider *Pisaura mirabilis* is a common species that lives in meadows, deciduous forests, and abandoned grasslands. In Central Europe, spiderlings hatch in the summer and reach maturity in the spring of the following year [48]. Mature males can be found up until June (summer), and females live longer during the summer. Males catch prey, wrap it in silk, and go searching for a female [35,38]. The male approaches the female, who then seizes the gift with her chelicerae [49]. Subsequently, the male inserts his pedipalp, which is an appendage that is modified in males for mating, into the female’s epigyne, the external opening of her reproductive tract, transferring sperm while the female consumes the gift [35]. The exact timing of the occurrence of mating behaviour in the field remains largely unknown [35,36]. All experiments with this species were conducted during the daytime; however, the males observed in the laboratory were noted to produce nuptial gifts at night as well [50].

### 2.2. Field Survey

We examined lighting conditions in microhabitats with *P. mirabilis* spiders in an uncut field in Slovakia (48°23′ N, 17°34′ E) on 26 May 2022. We chose a windless, partly cloudy day with a temperature of approximately 20 °C. The survey was conducted between 08:00 and 10:00, a time when spiders outside of vegetation are easily visible due to their activity in response to sunlight. While we did not record mating pairs during this survey, our own observations indicate that *Pisaura mirabilis* mates in both open and vegetated environments (P. Prokop, pers. obs.). Although these spiders are active during the morning, this does not imply that they are strictly crepuscular; they also exhibit activity inside vegetation later in the day. We recorded natural illumination (i.e., natural light falling on the spider) using a Kimo LX100 lux meter (Chevry-Cossigny, France), which has a measuring range of 0.1 to 150,000 lux. This range is relevant for understanding spider vision, as many species are adapted to detect a wide spectrum of light levels in their environments. For instance, some spiders have specialised adaptations that allow them to perceive light in low-light conditions and even ultraviolet (UV) wavelengths, which are not visible to humans [51,52]. The lux meter was placed directly above each spider, and we recorded spider sex (male or female) during these measurements. We also repeated the same measurements inside the vegetation at the base of the plant. In the laboratory, we replicated contrasting light conditions based on our field observations, although these conditions may not perfectly match those in the field.

### 2.3. Laboratory Experiment

#### Rearing Conditions

From April to May 2022, a total of 100 male and 100 female subadult spiders were collected from various grasslands and small woods near Bratislava (48°04′ N, 17°05′ E) and Trnava (48°37′ N, 17°58′ E), Slovakia. Each spider was individually housed in a ventilated 0.3 L plastic jar containing wet cotton to maintain humidity. The jars were placed in a laboratory environment with defined conditions, including a controlled photoperiod (12:12 h light:dark cycle), a constant temperature of 20 °C, and humidity levels maintained at 85–90% [53]. While this setup was not a climatic chamber, it provided stable environmental conditions to ensure consistent experimental parameters. The spiders were sprayed with water daily and provided ad libitum feeding three times a week with dead house crickets (*Gryllus assimilis*), approximately one adult cricket per feeding. Of the initial sample, 92 males and 93 females molted into adulthood during May 2022. After the experiments ended, all the spiders were released near their original capture sites by June 2022.

### 2.4. Experimental Procedure

On days 10 to 12 post-moult, each male was anaesthetised with CO_2_, and body mass (measured to 0.0001 g) and maximum prosoma width (measured to 0.01 mm) were recorded using a digital electronic balance and a digital calliper, respectively. Males were manipulated by applying dental silicone (Ultradent TM Universal, Ultradent Products, Inc. 1-800-552-5512, South Jordan, Utah, USA) to either (i) the dorsal side of a male’s abdomen (silk-intact) or (ii) a male’s spinnerets (silk-blocked) following Anderson and Hebets [54]. We simulated daylight during the mating trials using 40 W white-light bulbs (4000 K) in the laboratory. Reduced visibility for the spiders during mating trials was achieved by using red 5 W bulbs, which would simulate darkness for wolf spiders [30,31]. The spectral sensitivity of *Pisaura mirabilis* has not been studied; however, we speculate that their ability to perceive different wavelengths of light, including red, is similar to that of wolf spiders due to their phylogenetic closeness [55]. This approach reflects the natural habits of *Pisaura mirabilis*, which typically occur both outside and inside vegetation (pers. obs.), where light conditions vary significantly. Recognising that light in the field varies dynamically with time of day, weather, and the spiders’ movement between microhabitats, we employed controlled laboratory conditions to isolate the impact of specific light levels on mating behaviour. In our experiments, mating pairs of *Pisaura mirabilis* were subjected to red- or white-light environments. The males were randomly assigned to four treatments: Silk-blocked × Red light (n = 15), Silk-intact × Red light (n = 30), Silk-blocked × White light (n = 16), and Silk-intact × White light (n = 28).

Each mating trial was conducted in a glass terrarium (30 × 20 × 20 cm). The starting time for trials was c. 08:00. The female was placed inside the terrarium and was allowed 10 min to habituate and construct drag lines. The male was then introduced, at least 10 cm from the female. Other males demonstrated courtship behaviour after 1–5 min, which included touching the females’ drag lines, trembling of the palps and abdomen, jerking the body, and rapid rubbing of the legs [39,56]. Once a male exhibited courtship behaviour, we placed a gift item (a freshly killed cricket nymph, *G. assimilis*, mean = 0.027 g, SE = 0.004, N = 70) near him. We recorded whether the male wrapped the prey with silk and the latency time until the prey was wrapped. Silk-blocked males also exhibited wrapping movements, which were recorded. The investment of males in the production of nuptial gifts was measured as the total time males spent wrapping gifts in silk (in minutes) because these variables significantly correlate with the total amount of silk produced [34]. Males can interrupt their wrapping behaviour if a female approaches, and in such cases, we stopped recording the wrapping time. Recording resumed when the male began wrapping again. Additionally, if a male was rejected by a female and later resumed wrapping, we recorded the duration of silk wrapping at that point as well. We also recorded whether copulation occurred or not, the latency time until copulation (defined as the time from when the male and female were introduced until copulation occurred, including any time spent on silk wrapping), the copulation duration, the number of male rejections by the female (scored when the female did not show a willingness to copulate with the courting male), and which sex was in possession of the gift after the mating. Five males did not respond to the presence of the female by failing to initiate courtship behaviour and were not included in the experiments. In one trial, the female cannibalised the male without copulation. This trial has also been removed from the analyses. All the experiments were conducted simultaneously in two laboratories under different light regimens: white light and red light. Temperature and humidity were kept consistent across both rooms to ensure that environmental conditions did not influence the results.

In addition to silk investment, we measured several other behavioural variables that could influence mating outcomes:

Gift wrapping: Males are predicted to wrap gifts more frequently under red light, as increased visibility in poor conditions may encourage this behaviour.

Latency until wrapping: Males are expected to wrap gifts more quickly, however, than natural gifts under red light due to heightened visibility.

Female mate rejections: Females are likely to reject males with blocked spinnerets more frequently under red light because unwrapped gifts may be less visible and attractive.

Occurrence of copulation: Copulation is predicted to occur less frequently for silk-blocked males under red light due to the low visibility of their unwrapped gifts.

Latency to copulation: Males with intact spinnerets are expected to have similar latencies to copulation regardless of visual conditions, while latencies for silk-blocked males should be delayed under red light due to low gift visibility.

Copulation duration: Copulation duration is expected to be determined by gift size rather than wrapping; thus, visual conditions and wrapping should not significantly influence this variable [46].

Gift retention after copulation: Silk-blocked males are predicted to retain gifts after copulation less frequently than intact males because they have less control over the gift compared to those producing silk [45].

### 2.5. Ethical Note

As this study was conducted with spiders, a formal ethical review of the experiment was not necessary. We aimed to minimise, however, any potential stress or harm that could be caused to the animals involved in the experiment. Spiders could walk freely in their cages and were manually handled only when necessary. We used CO_2_ anaesthesia to minimise stress when taking morphometric data and when treating their spinnerets. No spiders were sacrificed for these experiments. We released all the spiders back into the wild after the experiments.

### 2.6. Statistical Analyses

Male and female body mass and prosoma width were significantly correlated (Pearson correlation: r_87_ = 0.64 and 0.45, both *p* < 0.001, respectively). Body condition was assessed as the residual of regression of body mass on prosoma width [57]. Differences in illuminance between outer and inner vegetation were analysed using the Wilcoxon matched pairs test, as data were not normally distributed. Male silk-blocking (silk-blocked or silk-intact) and light conditions (red or white light) were defined as categorical predictors. The male and the female body condition and the total number of male rejections by the female were treated as covariates. Wrapping gift with silk, copulation occurrence, and gift retention after copulation were analysed with the Generalised Linear Model (GLM) with binomial dependent variables. Copulation duration was analysed with GLM with a normally distributed dependent variable. Most of the data deviated from normal distribution and could not be altered using log, Box-Cox, or square-root transformation. In these cases (latency until wrapping prey, latency until copulation, duration of silk wrapping behaviour, number of male rejections by the female), GLM with Poisson distribution of the data was used. All statistical tests were performed with Jamovi 2.4.11 [58].

## 3. Results

### 3.1. Field Survey

We surveyed 38 spiders (82% were females) sitting on leaves of stinging nettle (*Urtica dioica*), grass, and black horehound (*Ballota nigra*) (76%, 18%, and 5%, respectively). The mean illuminance on leaves with spiders was 71,552 lux (SE = 3870) (range: 17,000–119,000). The mean illuminance measured at the base of the plant was 1790 lux (SE = 338) (range: 113–8626). The difference in illuminance between the outside and inside of vegetation was significant (Wilcoxon matched pairs test, Z = 5.37, *p* < 0.001).

### 3.2. Wrapping of Prey Gifts

When presented with a prey gift item, 82 of 89 males (92%) seized it. Out of the males who took the gift, 29/30 (97%) and 25/28 (89%) of the silk-intact males under red and white conditions wrapped it, respectively. As expected, because of the treatment, none of the silk-blocked males under red (0/15) or white light (0/16) conditions successfully wrapped their prey with silk. However, most of them exhibited wrapping behaviour by attempting to wrap the prey, indicating that the behaviour was performed even in the absence of silk production. Wrapping the prey was only significantly affected by silk-blocking (Table 1) because silk-blocked males were unable to wrap the prey with silk. We measured the time males invested in performing the same gift-wrapping movements as silk-intact males. Neither light conditions, the interaction term, nor the male or female condition was found to influence the likelihood of wrapping the gifts by males (Table 1). The latency time to wrap the prey was shorter under white light than under red light, but the effects of silk-blocking and male and female body condition were not significant (Table 1). However, the interaction between light conditions and silk-blocking significantly influenced the latency time until wrapping the prey (Table 1). Silk-intact spiders showed no differences in latency time between lighting conditions (red, mean = 9.93 ± 11.1 min, N = 28, white, mean = 9.28 ± 6.46 min, N = 22). Silk-blocked males under red lights started, however, to wrap gifts sooner than under white lights (red, mean = 7.64 ± 6.25 min, N = 14; white, mean = 12.3 ± 12.8 min, N = 14) (Table 1).

**Figure 1:**
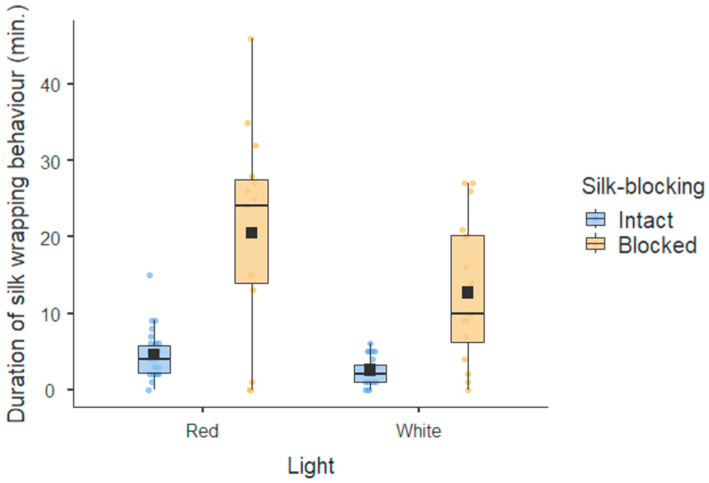
Male investment in gift production. The box is the interquartile range, and the whiskers indicate values that go beyond the 1st and 3rd quartiles. The line in the box is the median, and the dark square in the box is the average of the data set in each category.

Males under red lights invested more time in the duration of silk wrapping than males under white lights, regardless of whether they were silk-blocked or not (Figure 1). Silk-blocked males spent significantly more time trying to construct gifts than silk-intact males. The male condition was not associated with the duration of silk wrapping behaviour (Table 1). Males invested more time in silk wrapping behaviour when interacting with females in poor body condition. The more rejections from females the males received, the more time they invested in the duration of silk wrapping behaviour (Table 1).

### 3.3. Predictors of Female Mate Rejections

The number of female rejections of males was not influenced by silk-blocking, lights, male condition, or an interaction between variables, except for the female condition. Females in better body condition rejected males more than females in poor body condition (Table 1).

### 3.4. Copulation

Copulation occurred in 38/89 trials (42.7%). Neither the lighting conditions nor the male nor female conditions showed a significant effect on the occurrence of copulation (Table 1). Copulations occurred under red lights (23/45 trials, 51.1%) and white light conditions (15/44 trials, 34%) at similar percentages, although spiders tended to copulate more frequently under red lights. Silk-intact males (31/58, 53.4%) copulated significantly more frequently than silk-blocked males (7/31, 22.6%) (Table 1). Males received significantly fewer rejections in trials where copulation finally occurred (mean = 1.4, range: 0–6, SD = 1.5, N = 38) than when copulation did not occur (mean = 4.2, range: 0–15, SD = 2.5, N = 51) (Table 1).

The latency to copulation was not affected by light conditions, silk-blocking, or the male condition of the female; the more rejections the males received, the longer the latency time until copulation (Table 1). The latency to copulation was also positively associated with a greater investment in gift production by males (Table 1).

Copulation duration was influenced by the effect of treatment and the male condition (Table 1). Silk-blocked males (mean = 34.4, range: 5–112 min, SD = 37.6, N = 7) copulated for a significantly shorter time than silk-intact males (mean = 63.1, range: 14–134 min, SD = 32.1, N = 31). Males in better body condition copulated for a longer time than males in poorer body condition.

### 3.5. Gifts After Copulation

The gifts after copulation remained in 30 silk-intact and 6 silk-blocked trials. Females had gift remains in 19/30 silk-intact and 5/6 silk-blocked trials. None of the possible predictors influenced the likelihood that the gift would be monopolised by either sex (Table 1). A significant interaction term, Lights × Silk-blocking, suggests that silk-intact males monopolised gifts under red lights more than silk-blocked males, while no similar difference was found under white light conditions.

## 4. Discussion

Our results revealed that light conditions alter male investment in the production of nuptial gifts in *P. mirabilis*. The illuminance on leaves with sitting spiders was 40 times greater than the illuminance inside vegetation. *P. mirabilis* matings occur both outside and inside vegetation (P. Prokop, pers. obs.), suggesting that reproduction in this species is influenced by extremely variable visual conditions. Our principal hypothesis dealt with greater male investment in gift production in environments with low visibility. Using the total time that males spent wrapping prey with silk as a proxy for silk investment, we confirmed the hypothesis that males spent more time wrapping gifts under red lights compared to white lights. We suggest that red lights cause lower visibility of the gift, which is compensated for by adding more silk to enhance its visibility. Stålhandske [40] found that extra white gifts were accepted significantly sooner than natural or brown gifts, supporting the idea that the contrasting colour of the gift enhances its acceptance by the female. However, silk-intact males did not start wrapping the prey with silk sooner under red light than under white light, suggesting that light conditions alone do not promote faster silk wrapping. This idea is reinforced by the significantly greater effort of silk-blocked males who invest more time in gift production, particularly under red lights, despite their silk-blocked spinnerets not allowing them to do so. Alternatively, the fact that silk-blocked males spent more time trying to wrap the gift does not necessarily imply female involvement; it may simply reflect their efforts yielding no results.

An alternative explanation for the light condition hypothesis is that when the visibility is low, the female mate decision should rely on non-visual cues of male quality. For example, females of the wolf spider *Tigrosa helluo* use male chemotactile cues under red light more than under white light, which reduces female movement and aggression and finally increases mating success [30]. Similarly, the mate choice of female jumping spiders, courted in environments with low visibility, relies on vibratory rather than male visual displays [28,29]. *P. mirabilis* constructs nuptial gifts both nocturnally and diurnally [34,50] and is thereby prepared to court females under various lighting conditions. *P. mirabilis* females under red light may inspect gift quality based on chemical rather than visual cues [59]. We consider this explanation complementary to, rather than competing with, the light condition hypothesis because relying solely on chemical cues does not explain female readiness to accept brighter, rather than dull, nuptial gifts [40].

The importance of male silk during courtship was apparent when considering male mating success. Silk-blocked males copulated less frequently, and their average copulation duration was only half that of silk-intact males. Previous research failed to show that the absence of silk dramatically reduces male mating success [46]. Instead, our results suggest that the specific mating behaviour of silk-blocked males, which we discuss below, could be responsible for these results rather than the absence of silk per se.

The absence of silk could discourage females from accepting silk-blocked males due to the lack of chemical stimulants that entice females to copulate [59]. Shorter copulations could be caused by the lower control over the gift [45]. In fact, females mating with silk-blocked males had control over gifts in 83% of the trials, while females mating with silk-intact males took gifts in 63% of the trials. Although this difference did not reach statistical significance due to the smaller sample size of silk-blocked males, it is still informative. Additionally, wrapping a gift with silk extends the duration of female feeding [39], and the absence of silk in silk-blocked males could simply reduce female interest in feeding, consequently leading to reduced copulation.

Our second hypothesis posited that the success of the mating of silk-blocked males would be compromised, especially under poor visual conditions, as they will be unable to add silk to the gift, thus reducing its visibility. This hypothesis was not supported because the silk-blocked males were similarly successful under red light (poor visual conditions) as they were under white light (good visual conditions); the interaction term between these variables was not significant. The observed outcome appears to stem from a behavioural shift in silk-blocked males. These males spent a significantly longer time attempting to construct nuptial gifts, which consequently reduced the time available for courtship displays directed towards females. As a result, females may not have had sufficient opportunity to fully evaluate the quality of the (silk-deficient) gift under either visual condition. We suggest that females may not have had enough time to evaluate the quality of the gift under different visual conditions.

Statistical analyses showed that the effect of silk-blocking was relatively stronger than the effect of light manipulation. We propose that silk-blocking, in theory, prevents the male’s ability to shift from visual signalling to a different type of communication under red light (e.g., vibratory courtship) [60]. Silk-wrapping is performed by the male and is supposed to benefit the male rather than the female [59]. Alternatively, the absence of silk on the gift can make the gift of lower quality, and gifts are associated with genetic benefits for females [61,62,63].

Males invested more time in the duration of gift-wrapping behaviour when interacting with females in poorer body condition. Ježová et al. [56] found that the drag lines of hungry females stimulated males to produce gifts more than the drag lines of well-fed females. Although this result could be viewed as counterintuitive, given that well-fed, large females have greater reproductive potential in arthropods [64], hungrier *P. mirabilis* females are more sexually receptive [33,63,65]. Furthermore, this study showed that females in better body condition rejected males more frequently than females in poorer body condition. These results further support the idea that nuptial feeding evolved in the context of female foraging [34], particularly by males exploiting female foraging needs [65].

Previous research has demonstrated that *P. mirabilis* males in better body condition exhibit higher mating success compared to those in poor body condition [66,67]. Males in better body condition engaged in longer copulations than those in poorer body condition, which aligns with the findings of Albo et al. [66]. The male body condition was not found to be associated, however, with gift production [32]. These inconsistent findings may be attributed to the lack of experimental manipulation of the body condition in our study.

## 5. Conclusions

In conclusion, *P. mirabilis* lives and reproduces in vegetation, where light conditions vary greatly. However, this variable has never been considered in *P. mirabilis* research. Our results suggest that this spider is adapted to mate in both high- and low-light visibility, as mating success was not dependent on the type of light environment. The interplay between female visual and chemical perception seems to be affected, however, by low visibility, as males invest more effort in gift construction when visibility is low compared to when visibility is high. Future research should consider light conditions not only in the context of chemical and visual cues but also in relation to vibratory cues, which play an important role in spider intersexual communication. Crucially, research on the mating habits of *P. mirabilis* in the wild is essential to gain a deeper understanding of sexual signalling in spiders, particularly in the context of multimodal communication involving vibrational, visual, and chemical cues.

## Figures and Tables

**Table 1:** Results of GLMs exploring the impact of light regimen (red or white light) and male silk-blocking on mating success and mating interactions in *Pisaura mirabilis* in the laboratory.

Approach	Effect on	Variable	df	Test statistic (χ^2^)	Estimate	*p*
Binomial distribution	Wrapping gift	Whole model	5	96.1	-	<0.001
		Light	1	0.00000003	−0.65	1
		Silk-blocking	1	88.54	−24.41	<0.001
		Light × Silk-blocking	1	0.00000001	3.2	1
		Male condition	1	2.66	63.79	0.1
		Female condition	1	2.12	18.61	0.15
Poisson	Latency until wrapping gift	Whole model	5	19.3	-	0.002
		Light	1	7.44	0.2	0.006
		Silk-blocking	1	0.21	0.04	0.64
		Light × Silk-blocking	1	14.85	0.59	<0.001
		Male condition	1	0.03	−0.03	0.67
		Female condition	1	2.68	1.28	0.1
Poisson	Duration of silk wrapping behaviour (time)	Whole model	6	448	-	<0.001
		Light	1	39.67	−0.54	<0.001
		Silk-blocking	1	324.06	1.48	<0.001
		Light × Silk-blocking	1	0.099	0.06	0.75
		Male condition	1	0.3	1.37	0.59
		Female condition	1	6.21	−2.61	0.013
		Rejections by female	1	10.9	0.06	<0.001
Poisson	Female mate rejections	Whole model	5	8.09	-	0.15
		Light	1	0.62	0.1	0.43
		Silk-blocking	1	1.33	0.15	0.25
		Light × Silk-blocking	1	0.05	0.06	0.82
		Male condition	1	1.42	5.28	0.23
		Female condition	1	6.61	3.26	0.02
Binomial	Occurrence of copulation	Whole model	6	48.5	-	<0.001
		Light	1	3.16	−1.14	0.08
		Silk-blocking	1	5.33	−1.14	0.02
		Light × Silk-blocking	1	0.07	0.33	0.8
		Male condition	1	1.33	22.57	0.25
		Female condition	1	0.05	−1.54	0.83
		Rejections by female	1	32.6	−0.83	<0.001
Poisson	Latency to copulation	Whole model	7	111	-	<0.001
		Light	1	0.001	−0.003	0.98
		Silk-blocking	1	0.17	−0.05	0.68
		Light × Silk-blocking	1	1.46	0.26	0.23
		Male condition	1	1.11	2.98	0.29
		Female condition	1	0.28	0.59	0.6
		Rejections by female	1	69.1	0.18	<0.001
		Investment in wrapping gift	1	18.65	0.04	<0.001
Normal	Copulation duration	Whole model	7	13858	-	0.03
		Light	1	0.27	8.5	0.61
		Silk-blocking	1	12.68	−66.33	<0.001
		Light × Silk-blocking	1	0.28	−17.5	0.13
		Male condition	1	4.97	1057.6	0.03
		Female condition	1	0.92	−155.9	0.33
		Rejections by female	1	0.0009	−0.11	0.98
		Investment in wrapping gift	1	1.32	1.63	0.25
Binomial	Gift after copulation	Whole model	7	12.1	-	0.098
		Light	1	1.73	9.99	0.19
		Silk-blocking	1	2.4	11.14	0.12
		Light × Silk-blocking	1	6.2	23.65	0.013
		Male condition	1	1.24	50.23	0.27
		Female condition	1	1.23	17.75	0.27
		Rejections by female	1	3.32	−0.67	0.013

## Data Availability

The data supporting this study are included with the submission.
